# Climate-Driven Plant Response and Resilience on the Tibetan Plateau in Space and Time: A Review

**DOI:** 10.3390/plants10030480

**Published:** 2021-03-04

**Authors:** Prakash Bhattarai, Zhoutao Zheng, Kuber Prasad Bhatta, Yagya Prasad Adhikari, Yangjian Zhang

**Affiliations:** 1 Key Laboratory of Ecosystem Network Observation and Modeling, Institute of Geographic Sciences and Natural Resources Research, Chinese Academy of Sciences, Beijing 100101, China; pbhattarai2018@igsnrr.ac.cn (P.B.); zhengzt@igsnrr.ac.cn (Z.Z.); 2 University of Chinese Academy of Sciences, Beijing 100049, China; 3 Department of Biological Sciences, University of Bergen, N-5020 Bergen, Norway; kuber.bhatta@uib.no; 4 Department of Biogeography, BayCEER, University of Bayreuth, 95447 Bayreuth, Germany; yagya.adhikari@uni-bayreuth.de

**Keywords:** climate change, plant response, ecosystem functioning, species richness, plant traits, precipitation, temperature

## Abstract

Climate change variation on a small scale may alter the underlying processes determining a pattern operating at large scale and vice versa. Plant response to climate change on individual plant levels on a fine scale tends to change population structure, community composition and ecosystem processes and functioning. Therefore, we reviewed the literature on plant response and resilience to climate change in space and time at different scales on the Tibetan Plateau. We report that spatiotemporal variation in temperature and precipitation dynamics drives the vegetation and ecosystem function on the Tibetan Plateau (TP), following the water–energy dynamics hypothesis. Increasing temperature with respect to time increased the net primary productivity (NPP) on most parts of the Tibetan Plateau, but the productivity dynamics on some parts were constrained by 0.3 °C decade^−1^ rising temperature. Moreover, we report that accelerating studies on plant community assemblage and their contribution to ecosystem functioning may help to identify the community response and resilience to climate extremes. Furthermore, records on species losses help to build the sustainable management plan for the entire Tibetan Plateau. We recommend that incorporating long-term temporal data with multiple factor analyses will be helpful to formulate the appropriate measures for a healthy ecosystem on the Tibetan Plateau.

## 1. Introduction

### 1.1. Climate Has Been Warming on the Tibetan Plateau

Global air temperature has elevated by 0.74 °C (on average) over the past century, and it is predicted to increase by 1.5 °C by the end of 21st century [1]. Temporal trends in precipitation are rather erratic but have generally increased, especially in the mid-latitude land areas of the northern hemisphere. The Tibetan Plateau, one of the most critical and unique ecosystems of the world, has warmed by 0.42 °C decade^−1^ [2], and the warming rate in recent decades over the Tibetan Plateau has exceeded the averages for the northern hemisphere [3]. The precipitation trend over the Qinghai-Tibetan Plateau (QTP) is spatially varied, with increasing trends for most of the regions, especially in the eastern and central areas, whereas the western areas exhibit a decreasing trend [4].

### 1.2. The Changing Climate Has Affected Alpine Ecosystems and the Effect Magnitude Varies with Scale

With continuously increasing temperatures, higher latitude and mountain ecosystems are considered as the most vulnerable ecosystems [5]. Predicting terrestrial ecosystems’ response to ongoing climate change is one of the main challenges for ecological scientists. Climate change exhibits heterogeneous spatial patterns, and its nature, intensity and frequency may be completely redistributed from one spatial scale to next [6,7]. Moreover, the land use change in natural ecosystems such as grazing, land degradation or fire, often exhibits complex confounding, compounding, counteracting interaction with the climate change at different spatial and temporal scales [8].

The extreme habitat conditions (low temperature stress, poor nutrient availability and growing season length) in the alpine grassland affect plant performance and reproduction. Increasing temperature impacts the alpine ecosystem by its profound influence on plant growth, phenology and functions across terrestrial biomes [9,10]. However, the magnitude of the global climate change impact within and among ecosystems is highly varied [11] and scale-dependent [12].

### 1.3. Individual Species and Community Response to Global Changes and the Underlying Mechanism

The response of a species to climate change is likely to be individualistic, in accordance with the species’ climate niche. Climate change initially has an impact on the physiological and morphological structure of individual plants that alters the population of the species (demographic dynamics) and causes mortality or species loss. The community structure might also change in response to climate warming with the addition of novel species or removal of existing species, and hence, species composition and the dominance of the species within the community may fluctuate [11,13]; however, species composition on the northern part of the Tibetan Plateau is precipitation-dependent [14]. This structural change in the community alters the ecosystem functioning (for example, dynamics of nutrient availability, water resources). Climate change, hence, might affect ecosystem functioning by its influence on individual traits and alters the relative abundance of these traits in the community [15,16].

Moreover, plant response to climate change alters species population genetics on a fine scale [17] that changes the species composition [18] and species richness [19,20] in the species assemblage. Variation on a small scale may alter the underlying processes, determining a pattern operating at a large scale and vice versa; both can affect and give feedback to each other [21]. Therefore, alteration in the physiological response of specific functional groups will be important in determining the ecosystem dynamics under current climate change [22]. For example, climate warming enhances physiological activities in grass species and increases the annual net primary productivity of the ecosystems. But warming alters the community assemblages by species loss [23] and changes species composition [24].

### 1.4. Why Is It Necessary for Us to Investigate across Scale Responses of Plants to Global Changes?

**Figure 1:**
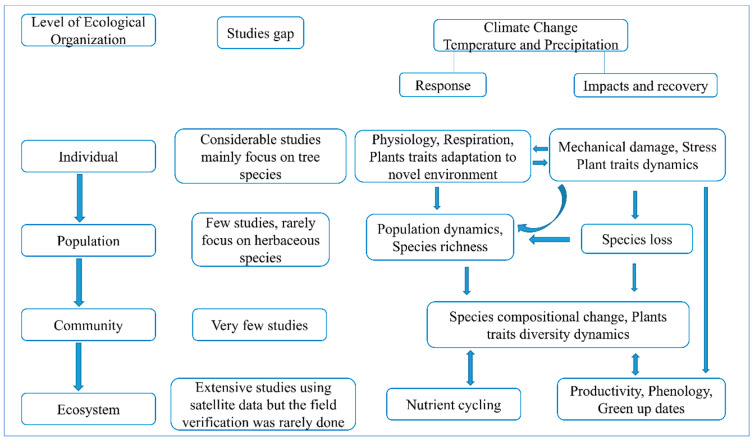
Conceptual diagram of the climate change impact and plant response across different ecological levels along with the research gap in the Tibetan Plateau. Initially, climate has a physiological or mechanical impact on individual plant species, which interact across multi-scale ecological organizations.

Similarly, disturbance in species composition and richness along with variation in individual plant traits (for example, physiology, growth) can regulate the ecosystem function, and such alterations are more sensitive to external drivers for potential response to regional environmental changes [25,26]. Thus, an understanding of the multiple scale interactions between cross-scale ecological organizations (from individual level to population or community or ecosystem, and from fine scale toward broad scale) helps us to improve our knowledge about the plant species responses to climate extremes (Figure 1).

**Figure 2:**
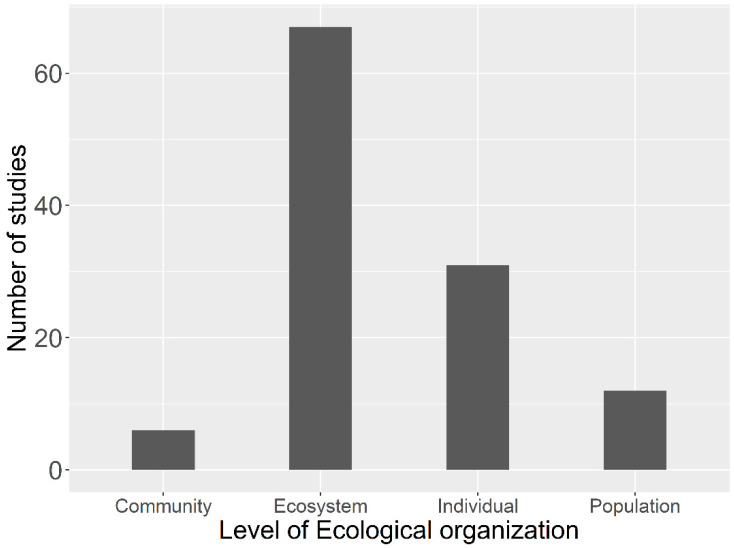
The number of studies that incorporate different levels of organization and their responses to climate change in the Tibetan Plateau. The studies that focus on more than one level get equal weight, adopted from Felton and Smith (2017) with modification. Studies were found and reviewed by searching the Web of Science and Google Scholar using the key words: climate change, species composition, normalized difference vegetation index (NDVI), productivity, Tibetan Plateau, precipitation, temperature. The list of citations and their DOIs are provided in the Supplementary Materials.

Studies conducted in the scaling of ecological organizations argue that scaling ecological processes on an individual level is complex [27]. However, recent studies [12,28] have scaled up the lower-scale (individual level) to large-scale ecosystem level processes (productivity or carbon/water cycling) with climate extremes. However, no attempts have been made so far to make a comprehensive review of the studies at different scale on time and space on the Tibetan Plateau (TP). The TP has a fundamental ecological significance, and reviews on cross-scale ecosystem responses to global change are scientifically critical. Here, we review the studies at different organizational scales (Figure 2) in both space and time conducted on the Tibetan Plateau to gain insight on studies that can be recommended for further research. The key finding of this review will be helpful for the preparation of a sustainable management plan for the entire plateau in order to sustain Tibetan Plateau health.

**Figure 3:**
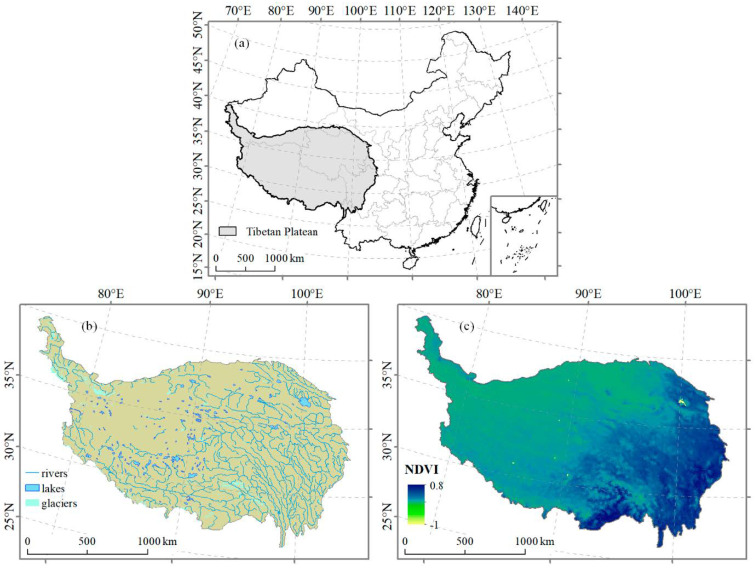
Map of the study area: (**a**) Location of the Tibetan Plateau in China, (**b**) distribution of rivers, lakes and glaciers in the Tibetan Plateau, (**c**) distribution of the normalized difference vegetation index (NDVI) in the Tibetan Plateau. The Tibetan Plateau stretches about 1000 km along the latitude and 2500 km along the longitude, with an average elevation exceeding 4000 m above sea level and an area of about 2.5 × 10^6^ km^2^.

The Tibetan Plateau (Figure 3), the “roof of the world,” has a unique climate and minimal anthropogenic influences, which makes it an ideal place to study the climate impacts on natural ecosystems [29]. The alpine grassland covers about 60% of the total area of the Tibetan Plateau and the remaining 40% includes desert and temperate forest ecosystems. These ecosystems have observed pronounced warming trends over the past three decades [30,31] with extensive ecological and environmental changes [32] acting as a small carbon sink [29].

Temperature- and precipitation-driven vegetation dynamics on the Tibetan Plateau have accelerated flowering and fruiting [31,33,34,35,36,37,38], increased vegetation greenness [37,38,39,40,41,42,43,44,45,46], increased productivity [47,48,49,50], increased ecosystem respiration [51,52], decreased species richness [20,24], increased glacier retreat [53] and thawing of permafrost [54]. However, these changes have varied spatially and temporarily with the water or energy differences.

A number of review studies have been conducted that include climate and cryosphere change [55], change in climate variables [56], environmental consequences [57], energy and water cycles [58], rangeland degradation [59], precipitation [60], and permafrost degradation [61], but how ecosystems respond to global changes across scales has never been summarized for the TP. In addition, comprehensive studies on plant responses to climate extremes at fine scale (individual species level) to broad scale (ecosystem processes) in space and time on the Tibetan Plateau are still lacking and need review.

## 2. Spatiotemporal Variation in Cross-Scale Ecological Organizations and Climate Indicators

### 2.1. Driving Factors on Spatial–Temporal Variations of Vegetation Dynamics on the TP

Cross-scale studies on ecological organizations (individual species to ecological process) vary spatially or temporally with climate indicators. There are multiple factors contributing to variations in spatial and temporal changes in species abundance, growth, community structure, community composition, NDVI, phenology and productivity in the Tibetan Plateau because of its geographical features as well as different responses of the complex physiological adaptation of alpine plant species [10]. However, the spatially heterogeneous temporal trends in climatic factors could explain the complex vegetation dynamics trend [44,62]. The spatial difference in the vegetation dynamics in the QTP is mostly related to water–energy dynamics at the regional scale.

#### 2.1.1. Driving Factors in Vegetation Dynamics on Ecosystems Scale

The variation in ecosystem level (NDVI, productivity and phenology) response in the TP is due to topography and unique vegetation response to climatic zones, which is attributed to temperature (energy) or precipitation (water) dynamics. At temporal scale, there is still debate on the overall NDVI and net primary productivity (NPP) dynamics in the Tibetan Plateau and the factors associated with these dynamics. Most of the studies (regional scale or local scale and remote sensing or experimental warming) on ecosystems levels showed the positive response with increasing temperature over the temporal scale. Productivity [63,64,65,66] and NDVI [44,45,67,68] increased with respect to increasing temperature.

The growing season NDVI in humid areas (mid and southeastern Tibetan Plateau) is closely related to rising temperature. However, the variation in NDVI trends on the southern Tibetan Plateau is elevation-dependent [68], where the average NDVI increased significantly from high-altitude alpine grassland toward low-altitude coniferous and broad-leaved forest [41]. The annual as well as seasonal NDVI of the eastern part of the southern TP increases more than that of the western part [42]. However, some studies showed that the annual NPP [69,70,71] and NDVI [62,72] remained constant on the northern Tibetan Plateau [73]. Decrease in overall productivity has also been observed on the entire TP [74,75], western Qinghai-Tibetan Plateau [65] and southwestern Tibetan Plateau [42]. Moreover, the southwest and south-central part of the TP have browning trends [45] at temporal scale, which is caused by a moisture deficit attributed to increasing evapotranspiration [76].

Regarding the phenological change, early onset was seen on the eastern edges of the plateau and delayed gradually toward the northwest [35,77,78]. The green-up dates were delayed in the southwest region [79] but advanced in the southeast and northeast [35,37] and central and eastern region of the plateau as well [79,80]. In most of the area in the southwestern part, spring warming delayed the start of growing season (SOS), whereas increasing precipitation advanced the SOS in most of the southeastern and northeastern plateau [37].

Spatial patterns of the phenological change in the Tibetan Plateau can be explained by the moisture and temperature gradient [77] as well as by the severe drying and cooling effect, which leads to a shrinking of the growing season length. The overall shrinking of the growing season length in the Tibetan Plateau can be explained by the corresponding climate condition, for example, soil temperature and moisture [77], permafrost degradation [61] and human activities [33].

#### 2.1.2. Driving Factors in Vegetation Dynamics on Individual and Population Scales

Spatiotemporal trends in the fine scale studies (individual or population level organization) also showed similar trends in impact and response to the climate indicators. Increasing temperature had a positive effect on the forest growth on the eastern Tibetan Plateau [2], northeastern TP [42,45,65], southern Xigaze, southwestern Qinghai province [65] and energy-limited alpine region of the southern TP [81,82], as well as on the humid southern part of the plateau [41,83]. Warming increased the overall vegetation greening and productivity. However, the negative impact of increasing temperatures was observed on moisture-limited areas of the Tibetan Plateau [84,85].

In contrast, increasing temperature had a positive impact on shrub recruitment till 1930 and a negative impact later [86] in the high alpine Tibetan Plateau. Shrub growth before 1930 was temperature-limited, and increasing temperature favored the seed recruitment by decreasing seed germination time [87], increasing the chance of seedling survival [88] and reducing frost damage and desiccation injury [89]. After 1930, the spontaneously increasing temperature crossed the optimal temperature for seed germination. Seedling growth along with warming-induced drought stress limited shrub regeneration [86,90].

Increasing temperature impacts directly at fine scale (individual level ecological organization) and the response trade is developed at individual level. Increasing temperature facilitates root growth and enhances photosynthesis. The continuously increasing temperature favors the seed production, increases nutrient availability, dispersal, germination, seedling establishment and lowers the tree mortality rate; this indicates a positive feedback between forest regeneration and temperature [81] and, thereby, subsequent tree establishment [91]. Warming may enhance the soil nutrient level and soil temperature, which is likely to be the limiting factor for plant growth and development in the alpine region [92].

On the other hand, strong radiation and increasing temperature might be expected to reduce the resilience of both treeline shrubs and trees by warming-induced drought stress on the entire Tibetan Plateau that may cause forest die-off. However, regional tree recovery is observed on the Tibetan Plateau by the diurnal temperature range dynamics; the greater the inter-annual increase in diurnal temperature, the higher the ability of tree recovery [93]. The reason might be because the forest species develop eco-physiological resilience against drought that maintains the vegetation greening and healthy ecosystem in the Tibetan Plateau, or the temperature may not reach the optimal temperature in most of the region of the alpine area of the Tibetan Plateau.

Therefore, both the temperature and available liquid water dynamics on the TP explain cross-scale vegetation dynamics over the entire region. However, the individual responses of cross-scale ecological organizations to climate indicators may provide detailed information on the role of water and energy in shaping vegetation dynamics on the Tibetan Plateau.

## 3. Individual and Population Level Responses to Climate Dynamics

### 3.1. Photosynthetic Rates Accelerate as the Function of Increasing Temperature

The photosynthesis activities in green plants initiate the process in which the resources enter the ecosystem. All plant species have unique ways to process and invest the resources, which have ample effects on species composition, species richness and ecosystem functioning [94]. Plants photosynthetic rates respond quickly to global change [95]. Climate impact on growing season length, particularly in temperature-limited regions of the TP, increases the leaf area index of the plant species [96,97,98]. Increasing the leaf area index increases the rate of leaf photosynthesis, which is widely used in leaf to ecosystem scale models. Leaf photosynthesis particularly depends on the maximum rate of Rubisco carboxylation capacity of RuBP. An increasing temperature accelerates the kinetics of carboxylating enzyme (Rubisco), favors oxygenation relative to carboxylation of RuBP [99,100] and increases photosynthesis rate. Thus, elevated temperature upsurges gross photosynthesis and autumn biomass in response [101]. Moreover, an elevated temperature increases apparent quantum yield or photosynthetic carbon gain [102] that increases the light utilization capacity to boost the photosynthetic rate [98,103]. Temperature beyond the optimal temperature retards photosynthesis rate [104], however, the TP is temperature-limited, and increasing temperature accelerates the photosynthesis rate. Warming-induced increasing photosynthesis rate has a growth response, increasing leaf area (leaf traits) or shoot elongation (shoot traits) manifested by an increase in cell number, cell size or both [98].

### 3.2. Species Traits Determine the Responses

Knowledge of plant traits and their distribution among the species and their response to specific drivers is useful for predicting community structural dynamics in an ecosystem. The root length traits and their dynamics are useful for explaining different plant groups’ response to drought. Drought reduces the aboveground biomass and promotes root growth. The decrease in plant growth and aboveground biomass under drought might be due to the physiological limitation on water transport [105,106]. The water stress increases the nitrogen concentration in the plant organs [107,108,109], and it is because of the accumulation of soluble protein during drought, which might be used later during the recovery period [110].

The shallow-rooted species, generally forbs, are more vulnerable to warming than the deep-rooted species [18]. Deep-rooted species operate to avoid potential functional damage by the accumulation of large belowground reserves that can tolerate the damage caused by heat stress or enhance susceptibility to freezing events [20,111]. Moreover, the ability of deep-rooted species to absorb the nutrients and soil water from the deeper soil zone make them more resistant to warming-induced drought. However, some species show different biomass accumulation and partitioning under different water regimes that increase water-use efficiency as a response to drought stress. Thus, drought can affect carbon assimilation and growth [112].

Increasing temperature modifies the leaf traits [113] (for example, warming increased leaf length, leaf size and specific leaf area), and plays a great role in the number of morphological and biochemical traits [98]. The phenological trait dynamics under climate warming illustrate the functional relationship of different species in the alpine plant community and the possible mismatch between the environmental drivers and, hence, explain plant communities’ responses to warming [114]. Increasing temperature advances the phenological events (bud break, flowering, and fruit coloring) and delays leaf senescence; however, the degree of the response varies among the species.

The negative impact of increasing temperature on leaf traits has also been observed. Warming-induced severe drought caused abnormal change in leaf ultrastructure, increased plastoglobules and swelled chloroplasts that damage photosynthetic activities and disturb carbon assimilation [112]. Moreover, the excess temperatures above species threshold retard photosynthetic rate [115] because of the variation in temperature-dependent chlorophyll content [116]. However, the stress-tolerance strategic responses with low growth rate that had been noticed on *Hippophae* spp. balanced inherent physiological processes under moderate drought stress by regulating N absorption and adjusting amino-acid profiles, which increased water use efficiency [112].

### 3.3. Species Reproductive Phenology

Global change strongly influences reproductive phenology (flowering and fruiting) of plants in alpine ecosystems of the TP. Climate impact can show significant effect on the duration of the reproductive cycle of plants (flowering and fruit development). Increasing temperature significantly shortens the reproductive stages among the species by shifting the phenological stages [117,118]. This causes the temporal overlap of reproductive stages in many individual species, and some late flowering species were unable to produce flowers and fruits [119,120]. This may shift the reproductive phenological patterns and change the species composition of the alpine ecosystem. The change in the reproductive phenology of alpine plant species was carried out by the water stress caused by warming [119,121], and the response depended on the time of flowering [18,119] and root traits [18,46] of the particular species. Delayed flowering event caused by water stress may make shallow-rooted and early flowering species more vulnerable to warming [121].

## 4. Community Responses to Global Changes

The climate indicators (temperature and precipitation) drive the biodiversity indicators (species richness, species composition or species diversity) in both plants [18,20] and animal communities [122]. Changes in plant species community structure, plant species composition, plant functional groups and morphological or physiological traits can regulate ecosystem functioning [123,124]. This internal change in community structure could be the proxy for predicting potential regional scale environmental changes [26,125]. However, the role of community structure and species composition on ecosystem functioning under climate change has been poorly understood [123].

### Community Structures and Species Composition Responses to Global Changes

Community structure can be defined as the unique number of species present in the community assemblages in a given environment and their interaction with each other. The impact of abiotic drivers on plant assemblage and the evolutionary interaction within the species in an assemblage are critical to understand the drivers and resistance of different plant groups to warming. Warming affects the microclimatic condition of the region, and continuous elevated temperature may exceed the plant tolerance capacity [20]. Under instant increase in temperature (in most of the warming simulation experiments), plant species may lack resistance to novel microclimatic conditions, and species disappear from the community assemblage [18]. The resistant traits of the species, in this case, can be used to understand temperature impact on the species.

The climate-induced resistance dynamics within the species in a community assemblage are responsible for species decline from the community. Increasing temperature in the Tibetan Plateau favors the growth and development of some species (mostly grasses) in the community assemblage, which may displace some forbs and sedges [20,126] because different species groups have exhibited different response patterns to global changes.

Warming-induced species richness decline in the alpine grassland of the Tibetan Plateau [127] indicates the rapid change in species composition. The variability in temperature and precipitation in different grassland types on the Tibetan Plateau determines the global impact on community structure and species composition. The increase in the grasses’ abundance positively correlates with the air temperature [128], whereas the abundance of sedges correlates positively with the soil moisture [121]. In addition, increasing nutrient availability from warming [129] favors more grass species [130].

Grass has the advantages of resource allocation because of inherent physiological capacity [131,132] and the elongated fall tiller [133]. The association of arbuscular mycorrhizal fungi with grasses [134] enhances the water intake and in turn absorbs elevated nutrients more efficiently [135,136], explaining the increasing abundances of grasses in the community. The increasing abundances and increasing height of the grasses from warming [135] might be responsible for the change of functional group.

Moreover, alpine plant species possess the adaptation competences with local abiotic condition. The environmental filtering effect on plant trait evolution and their diversity has a significant impact on the grassland productivity as well as on soil moisture availability [123]. Although physiological and morphological advantages help the grass to acquire more water, nutrients and light, plant communities with higher species richness and composition are more resilience to ongoing climate change [137,138]. Apart from this, change in species abundance and composition may change the trophic interaction between the species because of the species loss [139,140]. Change in species composition and species loss in the long run may thus potentially influence ecosystem structures and functions.

Thus, evolution of specific plant functional traits, inter- and intra-species relationships and their relative dominance dynamics, species richness, species composition and their stability should be considered at temporal and regional scales in studies on ecosystem functionality and sustainability of the alpine grassland of the Tibetan Plateau.

## 5. Ecosystem Processes Response to Climate Dynamics

**Figure 4:**
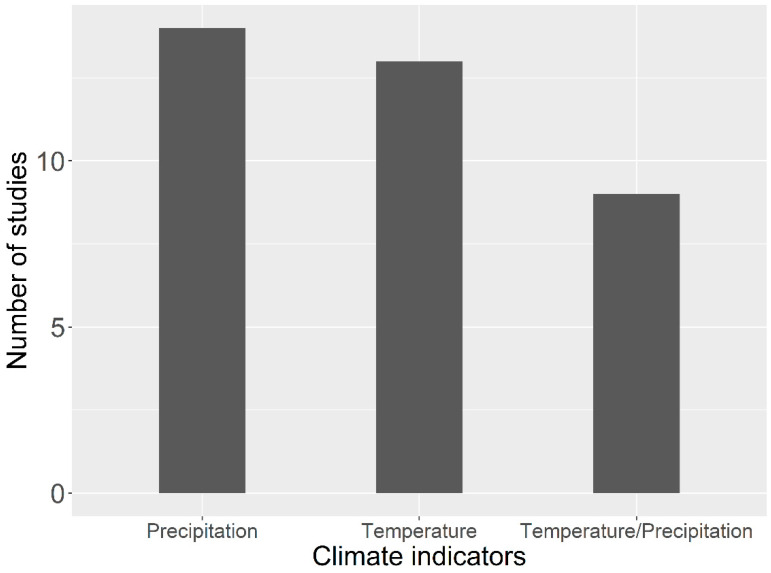
The number of studies indicating which climate indicators drive the ecosystem in the Tibetan Plateau. Both precipitation and temperature contribute equally to ecosystem functioning. The list of citations and their DOIs are provided in the Supplementary Materials.

Warming affects all the ecosystem types, ecosystem processes and organic matter pools at different rates and magnitudes, and the direction of ecosystem dynamics may vary on a spatiotemporal scale [141]. Ecosystem response to global change differs on a spatial scale, and magnitude of warming and other environmental factors (precipitation and nutrient availability) determine the direction and magnitude of carbon dynamics on terrestrial ecosystems. For example, warming on a tundra ecosystem accelerates carbon sources [142]; however, positive [143], negative [144] or neutral [145] response of plant biomass to warming has also been observed. Similarly, ecosystem dynamics in the Tibetan Plateau are not uniform, the geography of the plateau creates unique climatic variation within the plateau. Temperature and precipitation drive vegetation greening (NDVI), productivity and phenology at broad scale studies on the Tibetan Plateau and are considered in most of the studies. However, only limited studies have addressed the combined effect of temperature and precipitation on phenology and productivity in time and space (Figure 4). This suggests that ecosystem processes in the Tibetan Plateau are either energy-dependent or precipitation-dependent.

### 5.1. Phenology Responses to Global Changes

Phenology is advanced (early phenological cycles) on most of the part of the Tibetan Plateau in space and time. Phenological dynamics is considered as the major indicator of climate change that drives the ecosystem characters (for example, biomass accumulation, nutrient flow, production, pollination, etc.) [146]. However, consensus has been lacking regarding the role of temperature in phenological dynamics, i.e., advancement, static or delayed at temporal scale on the Tibetan Plateau.

The spatial and temporal phenological variation and the individual response to climate indicators caused the disparity in the result among the studies. Yu et al. [34] and Zhang et al. [80] showed that the phenological advancement in most parts of the Tibetan Plateau is because of the vegetation activity, greenness over space and climatic factors [147]. The advancement in the phenological change is primarily accomplished by the warmer spring and summer months, which tend to increase the photosynthetic activity of Tibetan grasses [34,101].

Temperature plays a more pronounced role in phenological change than precipitation on the Tibetan Plateau than in any other part of the world [147]; the rise in temperature advances the growing season [147] while the increase in soil moisture may delay the growing season onsets [148]. So there should be a counterbalance in plant phenology under warmer and wetter circumstances. In cold places, the increase in precipitation leads to delay in phenology. Jin et al. [77] suggested that the soil temperature and moisture information are far likelier to predict the phenological change in alpine grassland than air temperature and precipitation, which should have to be incorporated while phenology modeling.

However, Piao et al. [78] showed that there were no significant temporal trends in green-up dates on a regional scale in the Tibetan Plateau, despite continuous temperature increases. On the other hand, a study reported recent pronounced delay in green-up dates, which was attributed to the decrease in vegetation cover, apart from temperature and precipitation dynamics [149]. Therefore, factors like grassland degradation, thawing–freezing processes, climate warming and their combined effects should have to be incorporated for phenology shifts on the Tibetan Plateau [150].

### 5.2. Ecosystem Productivity Response to Global Changes

Studies conducted on both fine scale and broad scale in TP showed increasing productivity in recent decades due to increase in temperature [49,63,64,65]. However, some studies showed that the annual NPP of the Tibetan Plateau either remained constant [69,70,71] or decreased [74,75] with pronounced geographical heterogeneity in its trend.

Increase in temperature prolongs the growing season or increases nutrient availability, which in turn increases the productivity of the area. Warming may enhance the soil nutrient level and soil temperature, which is likely to be the limiting factor for plant growth and development in alpine regions [92]. Besides temperature, increasing precipitation has also explained the grassland NPP dynamics on the Tibetan Plateau during last five decades [65], mostly in the arid and semi-arid regions. Moreover, the productivity dynamics showed the increasing NPP trends from the northwest to southeastern TP because precipitation increases from northwest to southeast [151].

On the other hand, increasing temperature decreased NPP in the Qinghai-Tibetan Plateau, which was accomplished by moisture deficit due to increased evapotranspiration [76,152]. Piao et al. [65] revealed that the declining trends in the overall NPP of the QTP may arise with continuous elevated temperature associated with no or very less precipitation. However, Wang et al. [49] showed that the NPP of the grassland did not always increase with elevated warming and rising precipitation and decrease with increasing temperature and decreasing precipitation. They argued that precipitation was not the limiting factor in the southeastern QTP because the melting glacier increased the runoff in some river systems that provided enough water to terrestrial ecosystems [32]. The declining NPP in most of the area was also likely related to radiation; the region where precipitation and radiation decreased, and the reduction in energy input made the ecosystems energy-limiting despite the increasing temperature.

## 6. Response to Global Changes among Scales

Ecosystems’ responses to climate change dynamics and their resilience at fine or broad scale depend on the individual to community level response of the organism to climate extremes. An increasing number of “fingerprints” of climate change on different scales showed that species respond to climatic conditions with cascading effects through ecological organization [12]. However, it is not clear that to what degree the climate signals initiate the species responses through the three scales and the network within these scales. Climate impacts generally act independently on each scale, and species may evolve tolerance to global change [12], which can be visible at individual or population level but cannot be observed at ecosystem scale studies.

Moreover, most of the studies attributed ample evidence for phenological change to recent climate dynamics; however, plant communities are composed of many species, and these species do not response synchronously with global change [153]. The different responses of the plant species influence species interaction within an ecological network, which is only noticeable at individual level. For example, warming might advance spring phenology at the individual level, but this phenomena might not be obvious at ecosystem scale, because of the differences in the timing of multiple species responses to climate dynamics, which are affected by more confounding factors. In addition, the stress induced by climate extremes may cause mechanical damage or physiological adjustment at individual species level, which cannot be obvious from ecosystem level studies.

On the other hand, community structure, ecosystems and the ecosystem processes influence climate through multiple pathways [154,155], which can be observed in the broad scale studies (community and ecosystem scale studies) but not in the fine scale studies (individual and population level studies). According to the diversity–stability hypothesis, the community with a higher number of species tends to be more resistant to global change and helps to maintain the healthy ecosystem [156]; community level mechanisms of resistance can only be observed in community level studies but not at individual or ecosystems level.

Thus, the impact of global changes on plant physiological processes or morphology (fine scale) may alter population or community level process, and, in turn, can explain the upsurge or decline in ecosystem productivity. Here, the direct impact of global changes can only be observed in fine scale studies but not at ecosystem scale; however, the relative understanding among scales provides the knowledge on the relation of each scale to another. However, the direction and magnitude of responses vary within different levels of ecological organization. For example, the response magnitude is very fast at individual scale (physiology or recovery from mechanical damage) and slow at ecosystem scale (productivity or phonological change).

## 7. Concluding Remarks and Further Direction

Ecological studies that incorporate multi-scale ecological organizations are effective in solving the ecological problems of climate change at regional scales that determine the sustainability of the landscape. Our review showed that there has been very little research conducted on multiple scale studies on the TP.

The minimum anthropogenic activities in the Tibetan Plateau [157] reveal that ecosystem dynamics are mainly driven by climatic variables (especially temperature and precipitation). So, our review focuses on the impact of climatic factors on cross-scale ecological organizations, assuming that climate influences on vegetation dynamics in the Tibetan Plateau are more vigorous than anthropogenic influences. However, the impact of anthropogenic activities on plateau health cannot be neglected [158] and might need a separate synthesis of the published knowledge.

Increasing temperature (energy) accelerates the positive change in vegetation greenness, productivity and advances the green-up dates in most of the humid areas with abundant precipitation but retards with a decrease in regional precipitation, mostly in arid and semi-arid regions. The unknown precipitation trends on the Tibetan Plateau make it difficult for future predictions of plateau health that require studies incorporating long-term data. We noticed that most of the studies on temporal vegetation incorporate the data from the maximum 30 years period, which might not necessarily reflect long-term change in vegetation activity with respect to climate [44].

We are also aware of the complex characteristics of the physiography of the plateau, and that the evolutionary history of the plant species is related to such complicated landforms and geomorphic features. Thus, studies that incorporate long-term temporal data with multiple factor analyses will help to formulate the appropriate measures for healthy ecosystems in the Tibetan Plateau, a scientific basis for ecosystem management [159].

In our review, we also noticed that the ecosystem level studies (for example, NDVI, productivity or phenology) contradict to some extent the response toward the climatic parameters. Moreover, the results from the studies based on direct field observation (for example, productivity in the warming-simulated experiment) differ from the studies based on remote sensing satellite data (regional productivity). These contradictions and differences might be attributed to the methodological differences used in the studies. For example, most of the studies took into account only the growing season NDVI, while others dealt with the NDVI throughout the year. In addition, the calculated NDVI also varied with the sensor used in the studies. AVHRR NDVI decreased from 1998 to 2006, while MODIS and SPOT NDVI showed a slight increase during the same period in the steppe area of the Tibetan Plateau [85]. Moreover, the observed mismatch between the ecosystem dynamics and their associated factors may be due to the uncertainties in satellite-derived data. This demands a large number of ground observational validation data along with sophisticated approaches to scale up the data, ranging from individual to landscape level [160], which might be compatible with current-sized satellite-derived images [81]. Thus, further direction in ecosystem processes (NDVI, productivity and phenology) should be based on ground level observation to understand the ecosystem process and its associated factors.

## Data Availability

All data are available in the manuscript and the supplementary materials.

## Supplementary Materials

The following are available online at https://www.mdpi.com/2223-7747/10/3/480/s1, Table S1: The list of citations used for preparing the bar graph and their DOIs are provided in the electronic supplementary materials.
